# Heritability and Demographic Analyses in the Large Isolated Population of Val Borbera Suggest Advantages in Mapping Complex Traits Genes

**DOI:** 10.1371/journal.pone.0007554

**Published:** 2009-10-22

**Authors:** Michela Traglia, Cinzia Sala, Corrado Masciullo, Valeria Cverhova, Francesca Lori, Giorgio Pistis, Silvia Bione, Paolo Gasparini, Sheila Ulivi, Marina Ciullo, Teresa Nutile, Emanuele Bosi, Marcella Sirtori, Giovanna Mignogna, Alessandro Rubinacci, Iwan Buetti, Clara Camaschella, Enrico Petretto, Daniela Toniolo

**Affiliations:** 1 Division of Genetics and Cell Biology, San Raffaele Scientific Institute, Milano Italy; 2 Medical Genetics, Department of Laboratory Medicine, Institute for Maternal and Child Health IRCCS-Burlo Garofolo, Trieste, Italy; 3 Institute of Genetics and Biophysics”Adriano Buzzati-Traverso”, CNR, Napoli, Italy; 4 Institute of Molecular Genetics-CNR, Pavia, Italy; 5 MRC Clinical Sciences Centre, Imperial College Faculty of Medicine, London, United Kingdom; 6 Department of Epidemiology and Public Health, Imperial College Faculty of Medicine, London, United Kingdom; 7 Department of Internal Medicine, Diabetes & Endocrinology Unit, San Raffaele Scientific Institute, Milano, Italy; 8 Bone Metabolic Unit, San Raffaele Scientific Institute, Milano, Italy; Institut Pasteur, France

## Abstract

**Background:**

Isolated populations are a useful resource for mapping complex traits due to shared stable environment, reduced genetic complexity and extended Linkage Disequilibrium (LD) compared to the general population. Here we describe a large genetic isolate from the North West Apennines, the mountain range that runs through Italy from the North West Alps to the South.

**Methodology/Principal Findings:**

The study involved 1,803 people living in 7 villages of the upper Borbera Valley. For this large population cohort, data from genealogy reconstruction, medical questionnaires, blood, anthropometric and bone status QUS parameters were evaluated. Demographic and epidemiological analyses indicated a substantial genetic component contributing to each trait variation as well as overlapping genetic determinants and family clustering for some traits.

**Conclusions/Significance:**

The data provide evidence for significant heritability of medical relevant traits that will be important in mapping quantitative traits. We suggest that this population isolate is suitable to identify rare variants associated with complex phenotypes that may be difficult to study in larger but more heterogeneous populations.

## Introduction

Common complex traits are caused by multiple environmental and genetic factors each contributing to trait variability. Significant heritability and increased disease risk in relatives are measures of the importance of the underlying genetic factors; however, to predict the risk of a disease in healthy individuals requires detailed knowledge of the risk factors, their effect size and how they interact. The prediction of the genetic risk is still limited by the availability of common polymorphisms that are associated to the risk of a disease in the population. The large genome wide association studies (GWAS) performed to date have provided initial information on the genetic architecture of many diseases, but they have identified variants that individually explain a very small fraction of the genetic variance, to be used for an accurate prediction of the genetic risks [1], [2]. Isolated founder populations provide an attractive alternative for the study of complex traits as they typically exhibit greater genetic and environmental homogeneity than mixed outbred populations [3]. The origin from relatively recent common ancestors has increased linkage disequilibrium (LD) making these populations valuable tools for association studies. In addition, the availability of large genealogies makes linkage analysis a potent approach to identify disease loci. Several isolated populations have been described and have already proven useful to study Mendelian or complex traits [4]. Because of their history, geographical conformation and population admixture events that have taken place over the centuries, population isolates in Italy, might provide useful cumulative tools to unravel a significant part of the genetic diversity underlying complex traits [5], [6].

The population structure of a genetic isolate can be quite variable, from the very large populations of Sardinia [7] or Finland [8] to micro isolates such those described in the Cilento region [9], in central Sardinia [10] or in South Tyrol [11] in Italy. Genetic isolates can have a very ancient origin, as it is the case of Sardinia, or they may be quite recent as the Erasmus Rucphen family isolate that was founded in the mid 18^th^ century in the South West of the Netherlands [12]. Knowledge of the underlying genetic and population structure is essential to carefully design association studies, including choice of the most appropriate analysis approach that may depend from the degree of isolation, the length of the time the population has remained isolated and the size of the funding group [13], [14].

The aim of the present study is to characterize the population isolate of the upper Val Borbera, a large valley localized within the Apennine mountains of Piedmont, in the Northwest of Italy, geographically secluded from the surrounding areas. We present here evidence that the population, due to its isolation, high level of endogamy and lack of immigration in the last centuries, is indeed suitable for the study of complex disorders. As a part of a research program aimed at the identification of genes and variants associated to common disorders, we present here the analysis of a cohort of *1803* adult subjects from Val Borbera, selected based on their ancestry to represent the general population in the valley. We have studied a large set of quantitative traits that are relevant to major clinical domains, spanning from anthropometric measures to blood pressure and serum markers of diseases. The analysis of the heritability of each trait provides a quantitative assessment of the impact of the underlying genetic variation. Additional genetic correlation analyses suggest the presence of a set of genetic determinants that are likely to be relevant to multiple phenotypic traits. The results presented here are preliminary to the identification of genetic determinants underlying variation in quantitative traits, including risk factors for many common disorders.

## Results

### Cohort

**Figure 1:**
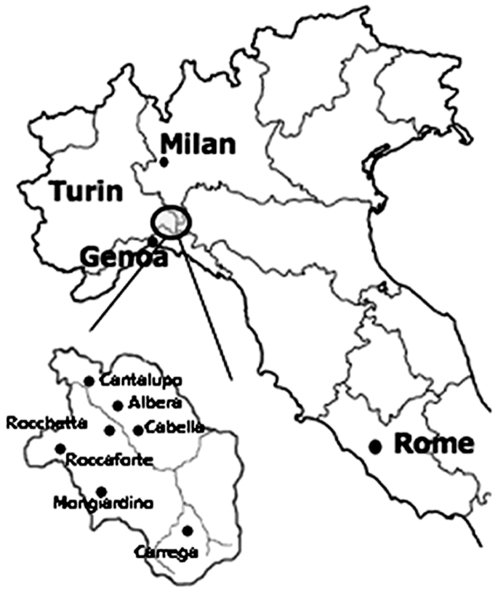
Geographical location of the Val Borbera in Northern Italy and position of the 7 main villages within the valley.

The Val Borbera is a large valley isolated from the surrounding areas by mountains and by a deep canyon on its western side. In the middle of 1800, the valley was inhabited by >10,000 people, living in seven villages located at about 400 to 800 m of altitude (Cantalupo Ligure, Albera Ligure, Rocchetta Ligure, Cabella Ligure, Carrega Ligure, Roccaforte Ligure and Mongiardino Ligure) in the Alessandria province (Fig. 1). Due to substantial emigration that occurred in the last century, the population descending from the ancient inhabitants, living in the valley or in the surrounding areas, now includes approximately 3,000 people.

**Figure 2:**
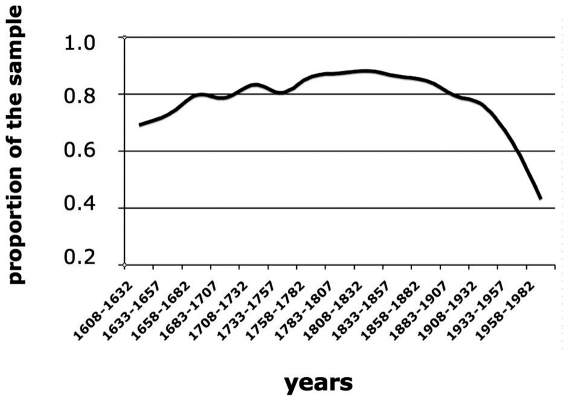
Percentage of endogamic marriages, over the centuries. 25 years periods were considered and are indicated along the X-axis. Marriages were counted from the marriage registers. Endogamy was calculated for the whole valley.

A complete genealogy of the population was reconstructed from birth, marriage and death records extracted from city archives starting from 1838 and from 17 (out of 24) parish church archives from approximately 1600. Data were collected from >96,000 records and the genealogy was reconstructed with a custom algorithm designed to manage the large number and assortment of different records from multiple sources across four centuries (Milani G et al., unpublished data). Most of the population (89.5%) was included in a large genealogical tree of >50,000 people tracing back up to 16 generations. The population size was determined from the number of births/25 years periods (Fig. S1). It seemed to increase in the 17th century and to remain stable until the middle of the 1800. It then underwent a fast expansion to decrease at around 1900 because of the emigration. Endogamy (Fig. 2) followed a similar trend: was 70% in the 17^th^ century, indicating some immigration. It increased sharply afterward, reached 80% with peaks of 90% in 1800 and started to decrease in the middle of the 1900.

**Figure 3:**
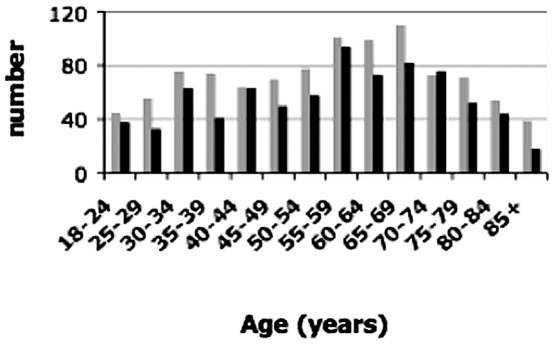
Age and sex distribution of the participants to the study: 5 years periods were considered. In black are the males, in grey are the females.

**Figure 4:**
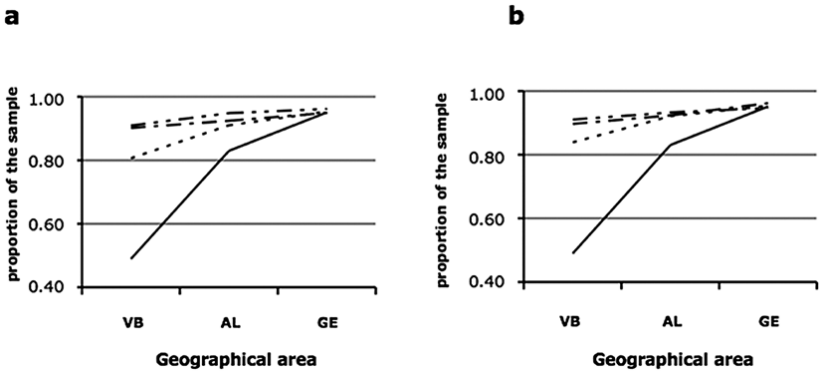
Birthplace distribution of the participants (continuous line), their parents (dotted line) and their grand parents (grandfather: one dot/line; grandmother: two dots/line) in progressively larger geographical areas: Val Borbera (VB) and the two surrounding provinces of Alessandria (AL) and Genova (GE). a: maternal lineage; b: paternal lineage.

A total of 1803 people aged from 18 to 102 years were enrolled in the study (Fig. 3). This sample was enriched in females (56%) and in older people (mean age 55 years). In our sample, individuals older than 65 years and 80 years represented 34.4% and 8.6% of the total population respectively, as compared to 29% and 5.8% in the 2001 census of the Alessandria province. About 50% of the participants were born in Val Borbera, >90% of the rest were born in the nearby area, within 25 km diameter from the upper Val Borbera (Alessandria and Genova provinces). Fig. 4a and 4b show the birthplaces of the maternal and paternal parents and grandparents of the participants; >80% of the parents and 90% of the four grandparents of the participants were born in Val Borbera.

Accordingly it was possible to link 1586 subjects into a large pedigree of 10,442 individuals (Genealogy 09_07_15) that included at least 16 generations ranging from 1500 to 1980. The average number of people in each generation, assuming 30 years intervals for each generation, was few hundreds individuals during the 17^th^ century, it increased to ∼1000 in the 18^th^ century and to 1500 in the middle of the 19^th^ century and decreased in the 20^th^ century due to emigration (table S1 in supplementary materials). The remaining 217 people that were not liked in the large pedigree were distributed in small families ranging from one large pedigree (7 individuals in a tree of 45 people in 7 generations) to 48 family trios, that could not be linked to the pedigree because of mssing data or poor quality of the genealogical information.

Using the full genealogy we calculated the kinship coefficient (kc) of the living descendants of the original population: the average kinship was 0.000373 with 3.5% of the entire population presenting a kinship >0. The average inbreeding in the population was 0.000746.

### Quantitative trait variation

**Figure 5:**
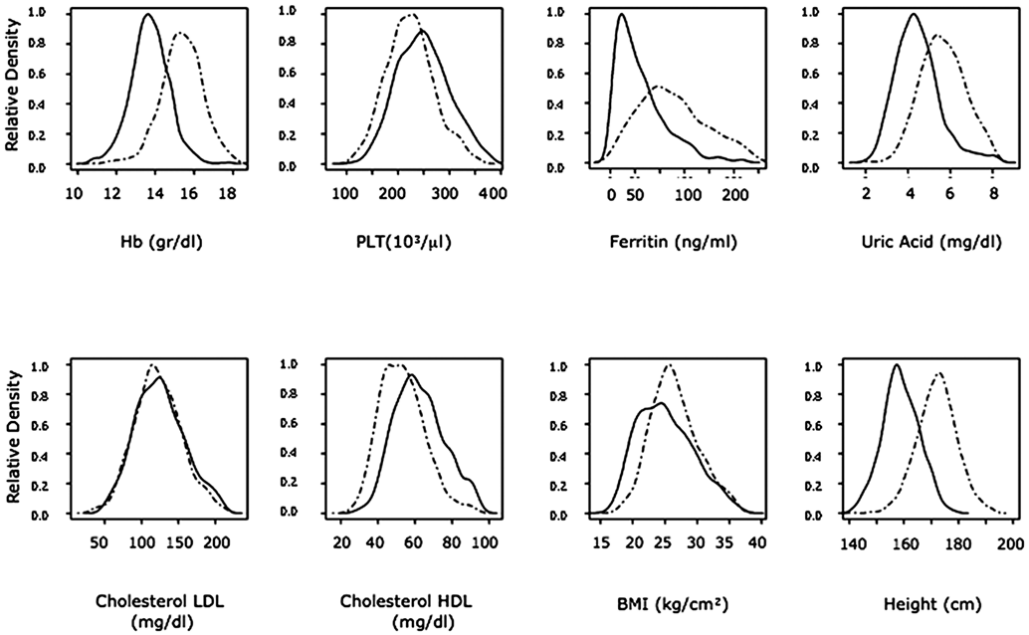
Distribution of eight representative traits in male and female participants. Relative densities were plotted for females (continuous line ) and males (dotted lines) for two blood cell measures (Hb and PLT), and for four serum (Ferritin, Uric Acid, Cholesterol LDL, Cholesterol HDL) and two anthropometric measures (BMI and Height). Abbreviations are as in table 1 and 2.

We collected and investigated 52 quantitative traits, including blood (n = 41), anthropometric (n = 4), cardiovascular (n = 3) and bone status, as determined by quantitative ultrasound (QUS) parameters, (n = 4) and assessed the effect of age and sex on each trait (Table S2–S4). As expected, most traits (47/52) presented significant differences in sex distributions (Fig. 5 and Table S2 and S3).

Further differences appeared when we compared the mean and standard deviations of all traits divided in four age ranges (18–45, 46–60, 61–74 and > = 75), each including approximately 25% of the sample (Table S4), as well as genders by age (Table S3).

### Quantitative trait heritability

The large sample size allowed us to accurately measure the heritability of each trait considered. First, a simple model with two variance components (an additive polygenic component and a individual specific environmental component) was used on data that were appropriately normalized to accommodate the assumptions of the model (see Material & Methods section). No major differences in the heritability estimated were observed between transformed and untransformed data. Three covariates (sex, age and the interaction of sex with age) were considered in all models. The results are shown in Table 1 and 2 and in Table S5 and S6. When sex was included in the model as a covariate, it explained 6.8% of the variance on average for blood test, 19.9% for anthropometric measures, 3% for the cardiovascular measures and 7.4% for bone status QUS parameters. When age was considered as a covariate, it explained a smaller proportion of the variance for blood tests (3.4% on average) and anthropometric measures (8.3% on average), but a higher proportion of cardiovascular and bone status QUS parameters (14% and 33.3% respectively). On average, the interaction between sex and age had an intermediate effect on heritability (Table 1 and 2).

**Table 1:** Heritability of blood measures.

Traits	p-value	p-value	p-value	Effect of covariates	Effect of covariates	Effect of covariates	Basic Model	Household Model	Dominance Model	
	sex	age	sex*age	sex	age	sex*age	H^2^	Narrow H^2^	Broad H^2^	p-valˆ
								(σ^2^_g+σ_^2^)	(σ^2^_g+σ_^2^_d_)/σ^2^	
Total Cholesterol	****	**	****	0.011	0.041	0.011	0.408	0.331	0.994	****
Cholesterol HDL	****		****	0.121	<0.001	0.013	0.618	0.618	0.618	
Cholesterol LDL			****	0.000	0.038	0.016	0.325	0.230	1.000	****
Triglycerides	****	****	****	0.027	0.066	0.025	0.424	0.385	0.737	*
Glucose	****	****		0.031	0.088	0.002	0.411	0.366	0.690	*
Uric acid	****	*	****	0.246	0.039	0.016	0.392	0.383	0.460	
Creatinine	****	****	*	0.269	0.022	0.002	0.333	0.310	0.493	
WBC	****	****		0.010	0.020	0.000	0.478	0.466	0.496	
Neutrophils	****			0.010	0.002	0.000	0.400	0.384	0.412	
Lymphocytes		****	*	0.000	0.067	0.004	0.409	0.409	0.409	
Monocytes	****	**		0.038	0.002	0.000	0.569	0.569	0.572	
Basophils				0.000	0.000	0.000	0.520	0.520	0.524	
Eosinophils	****			0.017	0.000	0.000	0.440	0.440	0.441	
RBC	****	****	****	0.211	0.011	0.032	0.595	0.595	0.595	
Hematocrit	****	**	****	0.300	0.001	0.011	0.484	0.479	0.551	
Hemoglobin	****	****	****	0.376	0.001	0.011	0.383	0.383	0.397	
MCH	****	****	****	0.076	0.015	0.010	0.571	0.570	0.571	
MCHC	****	****		0.132	0.033	0.000	0.464	0.464	0.461	
MCV	**	****	****	0.004	0.075	0.018	0.581	0.559	0.744	
PLT	****	****		0.072	0.042	0.001	0.598	0.591	0.680	
MPV		***		0.000	0.005	0.000	0.730	0.720	0.841	
Iron	****			0.041	0.000	0.000	0.240	0.240	0.240	
Transferrin	****	****		0.021	0.027	0.002	0.499	0.459	0.830	*
Ferritin	****		****	0.261	0.049	0.030	0.329	0.310	0.430	
Transferrin saturation	****		*	0.057	0.002	0.000	0.275	0.262	0.372	
ALT	****	****	****	0.087	0.000	0.036	0.325	0.304	0.464	
AST	****	*	****	0.047	0.017	0.030	0.297	0.272	0.499	
gGT	****	***	****	0.213	0.036	0.009	0.407	0.357	0.686	*
APh	*	****	****	0.000	0.164	0.026	0.592	0.592	0.592	
Total bilirubin	****			0.050	0.000	0.000	0.441	0.439	0.444	
LDH	*	****	****	0.006	0.090	0.019	0.477	0.430	0.860	**
CPK	****	****	****	0.074	0.004	0.035	0.378	0.374	0.400	
Total proteins	****	***		0.009	0.009	0.000	0.462	0.454	0.491	
Albumin	****	****	****	0.067	0.116	0.011	0.366	0.357	0.445	
IgA	****	****		0.012	0.063	0.000	0.505	0.470	0.742	
IgG	*	**	***	0.003	0.000	0.010	0.646	0.599	0.900	*
IgM	****	****		0.077	0.060	0.003	0.706	0.706	0.711	
Calcium	*	****	****	0.002	0.002	0.008	0.236	0.236	0.236	
Phosphorus	****	****	****	0.086	0.055	0.016	0.388	0.388	0.388	
TSH	****	****		0.006	0.047	0.000	0.369	0.361	0.385	
TPO-antibodies	****	***		0.042	0.027	0.002	0.279	0.262	0.388	

ˆ Polygenic and Household and Dominance Model were compared using Likelihood Ratio Test.

*p-value <0.05, **p-value <0.01,***p-value <0.001. ****p-value <0.0005.

Abbreviations: WBC: White Blood Cell; RBC: Red Blood Cell; MCH: Mean Corpuscular Hemoglobin; MCHC: Mean Corpuscular Hemoglobin Concentration; MCV: Mean Corpuscular Volume; PLT: Platelet Count; MPV: Mean Platelet Volume; ALT: Alanine Aminotransferase; AST: Aspartate Aminotransferase; gGT: Gamma-Glutamyl Transferase; APh: Alkaline Phosphatase; LDH: Lactate Dehydrogenase; CPK: Creatine Phosphokinase; TSH:Thyroid-Stimulating Hormone; TPO: Thyroid Peroxidase;

**Table 2:** Heritability of anthropometric, cardiovascular and bone status QUS parameters.

Traits	p-value	p-value	p-value	Effect of covariates	Effect of covariates	Effect of covariates	Basic Model	Household Model	Dominance Model	
	sex	age	sex*age	sex	age	sex*age	H^2^	Narrow H^2^	Broad H^2^	p-valˆ
								(σ^2^_g+σ_^2^)	(σ^2^_g+σ_^2^_d_)/σ^2^	
**Anthropometric Measures**										
Height	****	****		0.456	0.132	0.000	0.802	0.783	1.000	*
Weight	****	**	****	0.265	0.000	0.013	0.423	0.392	0.685	*
BMI	****	****	****	0.023	0.074	0.020	0.350	0.330	0.496	
Waist circ.	****	****	****	0.050	0.137	0.017	0.437	0.434	0.450	
**Cardiovascular Measures**										
SBP	****	****	****	0.024	0.351	0.022	0.257	0.256	0.257	
DBP	****	**	****	0.039	0.069	0.021	0.145	0.103	0.411	
Heart rate	****			0.028	0.000	0.000	0.258	0.258	0.254	
**Bone Density Measures**										
UBPI	****	****	****	0.010	0.481	0.011	0.353	0.289	0.987	**
Ad-SOS	****	****	****	0.000	0.530	0.012	0.265	0.164	0.946	**
Z score	*		****	0.004	0.065	0.013	0.276	0.209	0.654	*
T score	****	****	****	0.019	0.511	0.012	0.262	0.161	0.941	**

ˆ Polygenic and Household and Dominance Model were compared using Likelihood Ratio Test.

*p-value <0.05, **p-value <0.01,***p-value <0.001. ****p-value <0.0005.

"Abbreviations: BMI: Body Mass Index; SBP: Systolic Blood Pressure; DBP: Diastolic Blood Pressure; UBPI: UltraBound Profile Index; Ad-SOS: Amplitude Dependent Speed of Sound.

A wide range of heritabilities was observed for each group. All were statistically significant (p value from 1.26E-03 for Diastolic Blood Pressure (DBP) to 1.46E-62 for height) with standard errors ranging from 0.04 to 0.07 (Table S5). Considering all covariates, heritability ranged between 0.14 for DBP and 0.80 for height. Among blood tests, heritability ranged from 0.24 for calcium to 0.73 for mean platelet volume (MPV); among anthropometric measures, it ranged from 0.35 for body mass index (BMI) to 0.80 for height. It was around 0.26 for Systolic Blood Pressure (SBP) and heart rate (HR) (Table 1 and 2).

**Figure 6:**
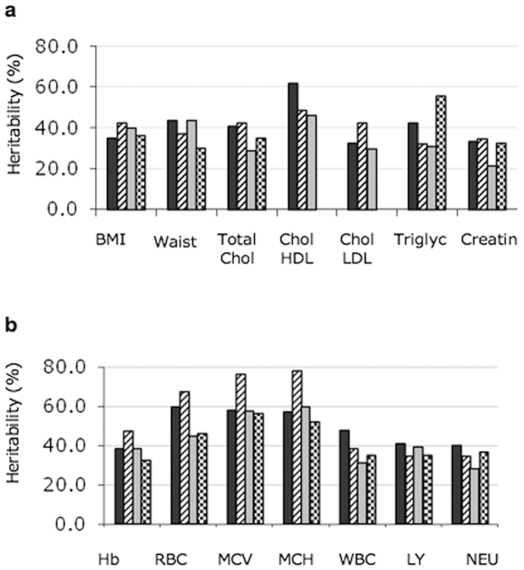
Comparison of the heritability of different genetic isolates from Italy. a. anthropometric and lipid measures. B: blood measures. The traits are indicated along the X-axis. Black bar: Val Borbera; dashed bar: Lanusei; grey bar: Cilento; checkered bars: Carlantino. Abbreviations are as in table 1 and 2.

We compared the heritability of each trait with other Italian population data either previously published (the population of Lanusei, in Sardinia [15]) or obtained from genetic isolates participating in the INGI (Italian Network of Genetic Isolates), namely the populations of Carlantino, a village in the South-East Apennines [16], and that of the Cilento area in the South-Western Italy [9]. BMI, waist circumference, height, glucose, uric acid and TSH (Fig. 6a and data not shown) had very similar genetic heritability in all populations. Significant differences were observed between the population of Lanusei and all the others for hemoglobin, RBC and red cell indexes (Fig. 6b), likely explained by the prevalence of thalassemia alleles in Sardinia [17]. WBC heritability was higher in Val Borbera and it resulted from higher heritability of the different blood components (lymphocytes and neutrophils are shown in Fig. 6b). Differences could be observed among isolates for triglycerides (higher in Carlantino), cholesterol HDL (higher in Val Borbera) and LDL (higher in Lanusei), while total cholesterol heritability was similar in all populations. (Fig. 6a). Transferrin heritability was very high (∼0.5) in Val Borbera when compared to two of the isolated populations considered here, ∼0.2 in Lanusei and 0.26 in Cilento, but also to other published data [12]. Other parameters of iron metabolism had similar heritability in all populations (not shown).

### Models with genetic dominance and shared siblings environment

We analyzed variance components that allowed for genetic dominance or shared sibling's environment (Table 1 and 2). The shared siblings environment effect was calculated from the narrow heritability, defined as the ratio of the trait's additive variance to its total variance. We detected significant genetic dominance and/or shared sibling environment variance components for several traits. Including genetic dominance, largely increased heritability, particularly for some traits. For total cholesterol and cholesterol LDL heritability increased from 0.48 and 0.32 to 0.99 and 1 respectively: on average, heritability for blood tests increased from 0.45 to 0.57. The same trend was observed for anthropometric measures with the average heritability increasing from 0.50 to 0.66 and particularly for the bone status QUS parameters, with an average increase from 0.29 to 0.88.

### Genetic correlation analysis

**Figure 7:**
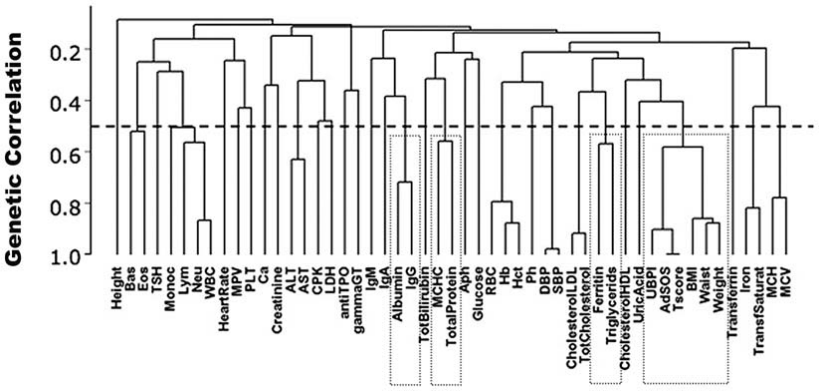
Clusters of genetic correlation. The 52 traits were classified into clusters inferred from genetic correlations between any two traits using the hclust function of R (http://www.r-project.org/). Genetic correlations are indicated on the Y-axis, with the highest correlations toward the bottom. Twelve values exceeded 50% correlation (table S7). Dotted squares highlight not expected genetic correlations. Abbreviations are as in table 1 and 2.

We calculated genetic correlation coefficients for all pairs of traits, as indication of common genetic determinants affecting phenotypic variation. This analysis showed 12 clusters of traits with substantial genetic correlation greater than 0.5 (Table S7). The correlations are shown in Fig. 7 grouped by means of a hierarchical clustering approach (see Methods). Traits connected by short branches share more of their genetic correlation than traits that join near the top of the tree. Some of the clusters occur because traits are correlated by definition as RBC, hemoglobin and hematocrit, MCV and MCH or iron and transferrin saturation. Others may be related by a common genetic background to be defined, such as BMI/waist/weight with the bone status QUS parameters UBPI, Ad-SOS and T score, ferritin with triglycerides or MCHC with total serum protein concentration.

### Familial clustering

**Figure 8:**
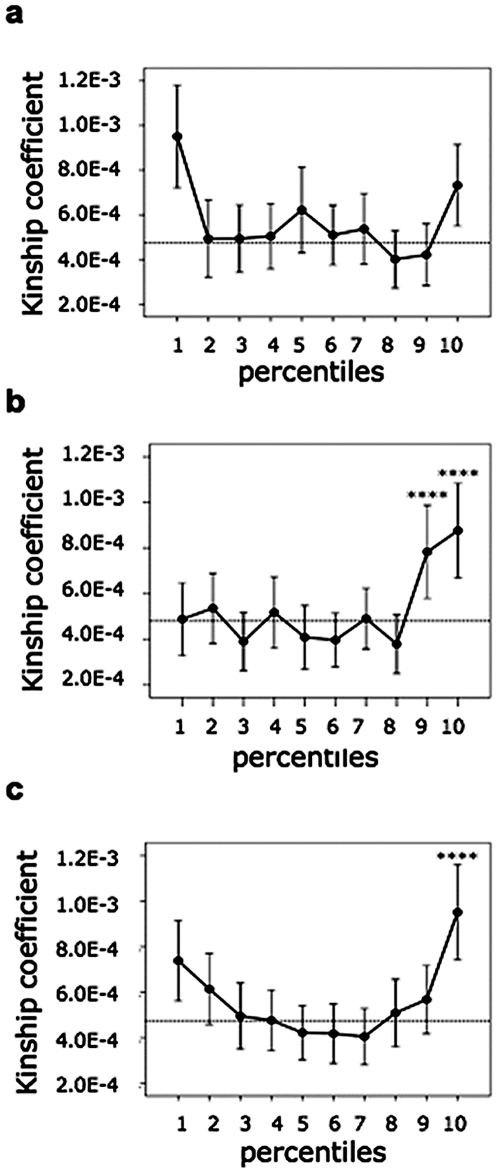
Clustering of extreme values for cholesterol HDL (a), triglyceride (b) and waist circumference (c): percentiles were constructed as described in the text, to account for the effect of sex and age. Kc was calculated within each percentile and the distribution was analyzed with ANOVA. Extreme percentiles were compared to the rest of the population and a p-value was calculated with the Mann-Whitney U Test: **** p-value <0.0005.

In isolated populations, rare variants characteristics of the population may be responsible for trait values at the extremes of the phenotypic distribution. They may identify familial clustering and higher kc in sets of individuals having phenotypic values within ten percentiles of the phenotypic distribution of the trait. We performed this analysis for some of the traits that define the metabolic syndrome (waist circumference, triglycerides, glucose, cholesterol HDL) [18] and BMI, all representing important risk factors for cardiovascular disorders. Since most of the variation was highly dependent from sex and age (Table S2 and S3), we considered the distribution of the residuals after correction for age and/or sex. As shown in fig 8a, significant enrichment in kc was detected among individuals at the 9^th^ (n = 163) and 10^th^ (n = 164) percentiles of the distribution of triglycerides: kc = 7.83E-4 versus 4.79E-4 for the rest of the populations and kc = 8.76E-4 versus 4.73E-4 (Mann-Whitney test p-value 7.07E-9 and 7.13E-9 respectively). Significant enrichments in kc was also found among individuals (n = 176) in the 10^th^ percentile of the waist circumference distribution (fig. 8b): kc = 9.51E-4 versus 4.76E-4 (Mann-Whitney test p-value 9.48E-9). Distribution of the kc among individuals at different cholesterol HDL percentiles (Fig. 8c) was overall significant (ANOVA between groups p-value 8.41E-05). Glucose distribution presented a higher kc at the lower extreme while BMI presented higher kc at both extremes (Mann-Whitney tests p-value 8.004E-10 for glucose; 2.3E-9 and 7.0E-3 for the 1° and 10° BMI percentiles, respectively) (not shown).

Analysis of the family clustering of the extreme phenotype groups pinpointed to large multigenerational families (not shown) that are expected to be enriched for rare and major-effect variants. These families may represent an informative set of individuals suitable for quantitative trait linkage analysis.

## Discussion

In this paper we report a demographic and epidemiological analysis of a genetically isolated population, settled in Val Borbera, a large valley in the North West Apennines, in Italy. Around 60% of the modern descendants, still living in the 7 main villages of the valley or in the nearby areas, have been recruited based on their ancestry. Analysis of the large genealogy constructed starting from city and parish archives showed not only that endogamy was high in the past, but also that >90% of the participants to the study had 4 grandparents born in the valley and that 87% were connected in a unique large genealogical tree that included up to 16 generations tracing back to the 16^th^ century.

We collected information on a large number of traits, including blood, cardiovascular, anthropometric and bone status QUS parameters. Most of the trait distributions presented large differences between age groups and sexes, in keeping with similar data from the general outbred population, and further confirming that genetic isolates are good representation of the general population.

The size of the cohort and the availability of a genealogy provided the opportunity to measure accurately the heritability of many traits, to compare the effect of age and sex as well as to look for genetic determinants shared by different traits. Overall, trait heritability was on average higher for blood measurements and for anthropometric traits and lower for cardiovascular and bone status QUS parameters. These results were consistent with previous studies [15], [19] and with those obtained by similar analysis in other genetically isolated populations from other italian areas, such as Sardinia, South West (Cilento) and South East (Carlantino) Italy. The heritability for haemoglobin and erythrocyte indexes, higher in Sardinia *vs* the other isolates, was clearly related to the significantly higher prevalence of alpha and beta thalassemia alleles among Sardinians as compared to continental Italy [17]. Similar differences were found for other blood and lipid measures, suggesting population-specific genetic components for some of the traits. In addition, specific traits showed evidence for genetic dominance, as previously reported for other populations [15], [20].

The analysis of genetic correlation coefficients showed overlap of genetic determinants for multiple traits. Many of the traits that shared genetic components were related, confirming the power of the analysis. However, other associations (indicated by a dotted line in Figure 7) were less obvious. We only partly confirmed the overlap of the traits of the metabolic syndrome (glucose, HDL cholesterol, triglycerides, waist circumference and blood pressure) [15], but in our cohort we found common genetic components for anthropometric measures (BMI, waist circumference and weight) and bone status QUS parameters. Some of these findings are in agreement with previous observations such as the controversial issue of metabolic syndrome and osteoporosis [21] and a recent report that higher bone mass density in the metabolic syndrome is largely determined by abdominal obesity [22]. Of note, we observed that triglycerides clustered with ferritin. This novel finding could be related to the occurrence of hyperferritinemia, in some cases with moderate iron overload, in subjects with metabolic syndrome [23], [24]. The observation provides the first suggestion of a shared genetic component between these parameters and points to a common genetic background for the two traits.

Overall the data presented here indicate that the Val Borbera population cohort may provide a good setting for identification of genetic factors controlling variation of clinically important quantitative traits and/or risk factors of disease not only by population-based association studies, but also by linkage analysis. Using the available genealogical information, power simulation for the whole pedigree resulted in 80% power to detect a significant lod score > = 3 with a trait heritability of ∼0.28 (Fig. S2). We also showed that in this cohort it may be feasible to identify rare variants that may contribute substantial insight into the genetic architecture of common diseases [2]. We could show that individuals presenting extreme values for related traits, namely the metabolic syndrome component traits and BMI also presented higher kc compared to individuals in other percentile groups and in most instances also to the other groups considered together. Many of the individuals and the extreme groups could be clustered in large multigenerational families presenting homogenous phenotypes. Analysis of such families may allow to identify new loci and particularly rare, high impact variants, that may largely contribute to the total disease risk and could account for a larger part of the heritability of a given trait [25].

In conclusion the Val Borbera cohort appears suitable for identifying genes involved in variation of medically important quantitative traits. As the range of human variation extends across most of the human populations, the study of such relatively small cohort and of the other isolated cohorts from Italy participating in the INGI, should be relevant to the genetic characterization of the human populations and particularly to identify rare variants that may represent a relevant disease risk factor in the general population.

## Materials and Methods

### Population recruitment

Inhabitants of the valley were invited to participate in the study by public advertisements through local authorities, televisions and newspapers as well as local physicians and mailings. Meetings were organized in all villages to present the project and its aims. The importance of the participation of entire families was underscored in all instances, nevertheless all people that volunteered to participate were included in the study, providing they had at least one grand parent from the valley. The study, including the overall plan and the informed consent form was reviewed and approved by the institutional review boards of San Raffaele Hospital in Milan and by the Regione Piemonte ethical committee.

Only individuals aged 18 years or older were eligible to participate. Each participant signed the informed consent. Clinical data and anthropometric measurements were collected by a team of MDs from the San Raffaele Hospital and by a local nurse. A standardized questionnaire was used to collect family and personal history. Blood pressure was measured with a mercury sphygmomanometer. SBP and DBP were the average of four measurements done with subjects in a seated position in a quiet environment. Heart rate was recorded by 12-lead electrocardiography. Fasting blood samples (about 20 ml) were obtained in separate sessions, in the early morning. Blood was tested the same day or aliquoted and stored for further analysis.

A standard battery of tests (see Table S2) was performed in the laboratory of ASL 22 - Novi Ligure (AL). Thyroid peroxidase (TPO) antibodies were measured from frozen serum samples by a radioimmunoassay using a commercial kit (Medipan, Berlin, Germany).

Bone status measurements were done with the DBM Sonic Bone Profiler (Igea, Italy), which follows transmission of an ultrasound beam through the four phalanges of one hand. Data was transferred and stored anonymously in a MySQL database.

#### Quantitative Traits Variation Analysis

SPSS 17.0 (SPSS, Chicago, IL, USA) and in-house R-2.8.1 scripts (The R Project for Statistical Computing [http://www.r-project.org]) were used for descriptive and inferential statistics analyses. Non-parametric testsd were used to investigate significance of the differences among independent, not normally distributed values. Gender differences were assessed by the Mann-Whitney U test, whereas the medians among ranges of age were compared using the the Kruskal-Wallis test.

### Heritability analysis

A maximum-likelihood variance components analysis was used to calculate the proportion of the overall variability due to the single covariates [26]. A rank-based transformation method called *inormal*, using an inverse normal transformation as performed by SOLAR (http://solar.sfbrgenetics.org/), was used to deal with kurtosis and skewness and to normalize each trait distribution.

A basic model was used to estimate the additive polygenic component σ_a_^2^ and environmental component σ_e_^2^ of each trait variance due to mean effects of single alleles. Polygenic model as performed by solar (http://solar.sfbrgenetics.org/) quantified heritability as h^2^ = σ_a_^2^/σ_a_^2^+σ_e_^2^ and provided an estimate of the degree to which the offspring phenotypes are explained by parental phenotypes.

Further models were used to estimate additional variance components as genetic dominance σ_d_^2^ and sibship effect σ_s_^2^. In dominance model, variability of each trait is explained by Var(T_i_) = σ_a_^2^+σ_e_^2^+σ_d_^2^ and the covariance between values for each pair of individuals as Cov(T_i_, T_j_) = 2ϕ_i,j_σ_a_^2^+Δ_ij_σ_d_^2^ where Δ_ij_ represents the probability that individuals i and j share two alleles identical by descent (IBD) based on their kinship coefficients. Genetic dominance model estimated heritability in broad sense as H^2^ = σ_a_^2^+σ_d_^2^/σ_a_^2^+σ_e_^2^+σ_d_^2^. In household model the excess of similarity between siblings may be attributed to common early childhood environment and the variance is defined by Var(T_i_) = σ_a_^2^+σ_e_^2^+σ_s_^2^ and the covariance as Cov(T_i_, T_j_) = 2ϕ_i,j_σ_a_^2^+I_sib(i,j)_ σ_s_^2^ where I_sib(i,j)_ corresponds to 1 for full siblings and 0 for other pair of individuals on genealogy. Sibship effect model estimated heritability in narrow sense as h^2^ = σ_a_^2^/σ_a_^2^+σ_e_^2^+σ_s_^2^.

### Bivariate trait analysis

A base polygenic model as developed in GHOST 0.0.9 (http://www.sph.umich.edu/csg/chen/ghost/) was used to estimate the heritability of each pair of traits, to extract a genetic ρ_g(Y,Z)_ and an environmental ρ_e(Y,Z)_ correlation coefficient for each pair of traits [27].

A dissimilarity matrix, reported |1–ρ_g(Y,Z)_| for all pair of traits, was used as input for the R function hclust (http://www.r-project.org/) to create a dendrogram object with an average agglomerative hierarchical method of clustering which connects trait by trait the most similar ones.

### Familial clustering of traits

Using SPSS, each trait value was fitted by a linear regression curve that considers all significant covariates to estimate the expected values for each individual. The differences between observed and predicted values were calculated for each individual to obtain residual values of each trait considered. All individuals values were divided in percentiles and individuals in every percentile were submitted as input for KinInbcoef 1.0 (http://www.stat.uchicago.edu/~mcpeek/software/CCQLSpackage1.3/) to calculate kc for each pair, based on the genealogy [28]. Kc distributions were compared using ANOVA and individuals with extreme residual values were compared with kc calculated among all the other individuals (Mann-Whitney U Test/Median Test by SPSS). Individuals with extreme residual values for each trait, which showed the significant highest kinship distributions, were used to investigate familial clustering with Jenti [29].

## Supporting Information

Figure S1 Number of births from the 17th century to recent times. Birth acts over 25 years periods were considered and are indicated along the X-axis. As also shown by the endogamy curve (Fig. 2), an increase in the number of births is visible at the beginning of the 17th century, suggesting immigration and increase in the population size.
(0.22 MB TIF)

Figure S2 Power calculation of significant lod scores that could be obtained with the whole pedigree for traits of different heritability. Continuous line: lod score> = 3, broken line: lod score > = 2. The analysis was done with Solar (http://solar.sfbrgenetics.org/).
(0.11 MB TIF)

Table S1 individuals/generation.
(0.03 MB XLS)

Table S2 Distribution of the traits.
(0.03 MB XLS)

Table S3 Mean values of the traits.
(0.05 MB XLS)

Table S4 Age ranges.
(0.05 MB XLS)

Table S5 Heritability parameters.
(0.05 MB XLS)

Table S6 Effect of each covariate on heritability.
(0.03 MB XLS)

Table S7 Clusters of traits above o.5 cutoff of Genetic Correlation.
(0.03 MB XLS)
