# Supplementary figures and images for: Heritability and Demographic Analyses in the Large Isolated Population of Val Borbera Suggest Advantages in Mapping Complex Traits Genes
Source: PLoS One. 2009 Oct 22;4(10):e7554. doi: 10.1371/journal.pone.0007554 (PMC2761731; doi:10.1371/journal.pone.0007554)

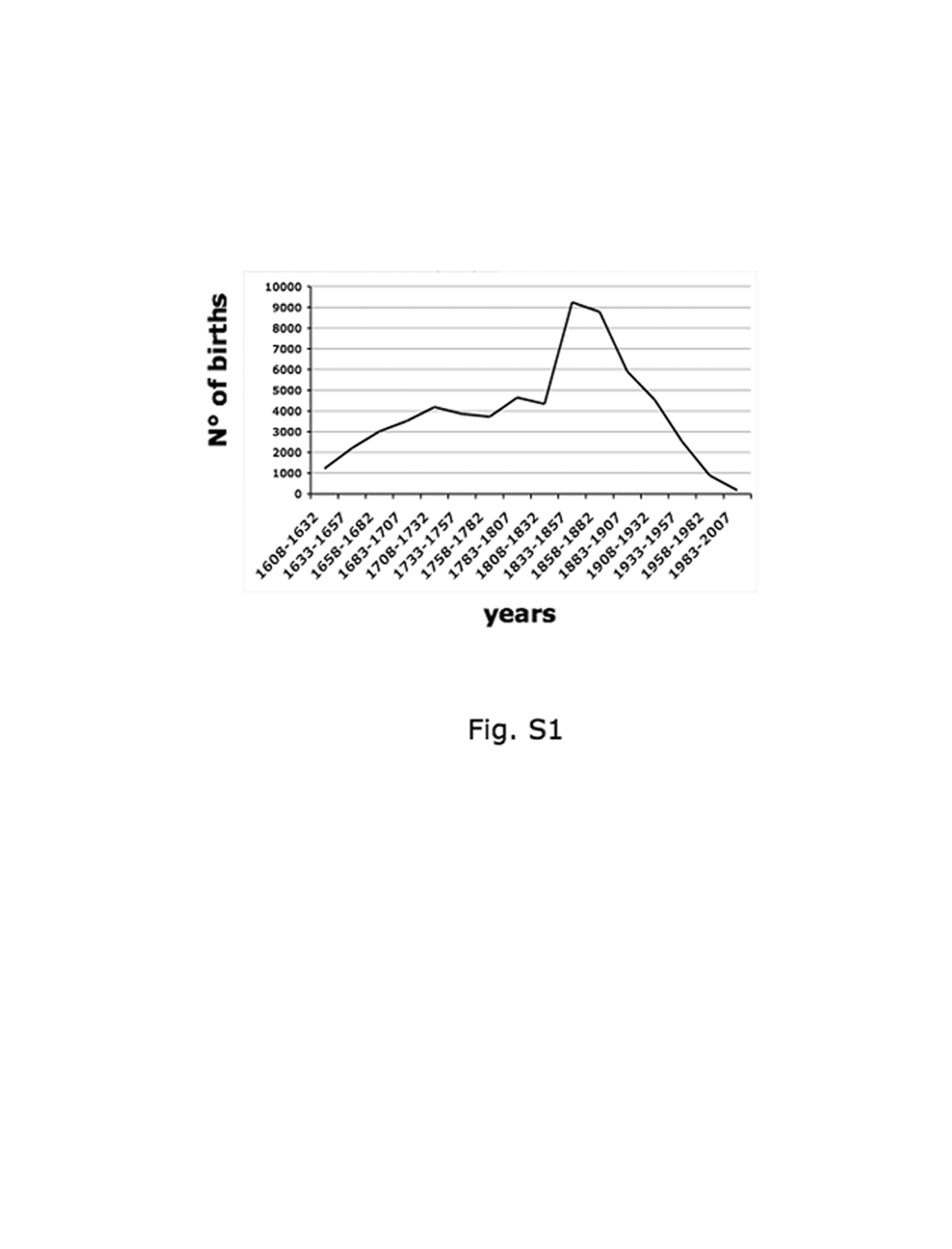

Supplement: Figure S1 — Number of births from the 17th century to recent times. Birth acts over 25 years periods were considered and are indicated along the X-axis. As also shown by the endogamy curve (Fig. 2), an increase in the number of births is visible at the beginning of the 17th century, suggesting immigration and increase in the population size. (0.22 MB TIF) [file pone.0007554.s001.tif]

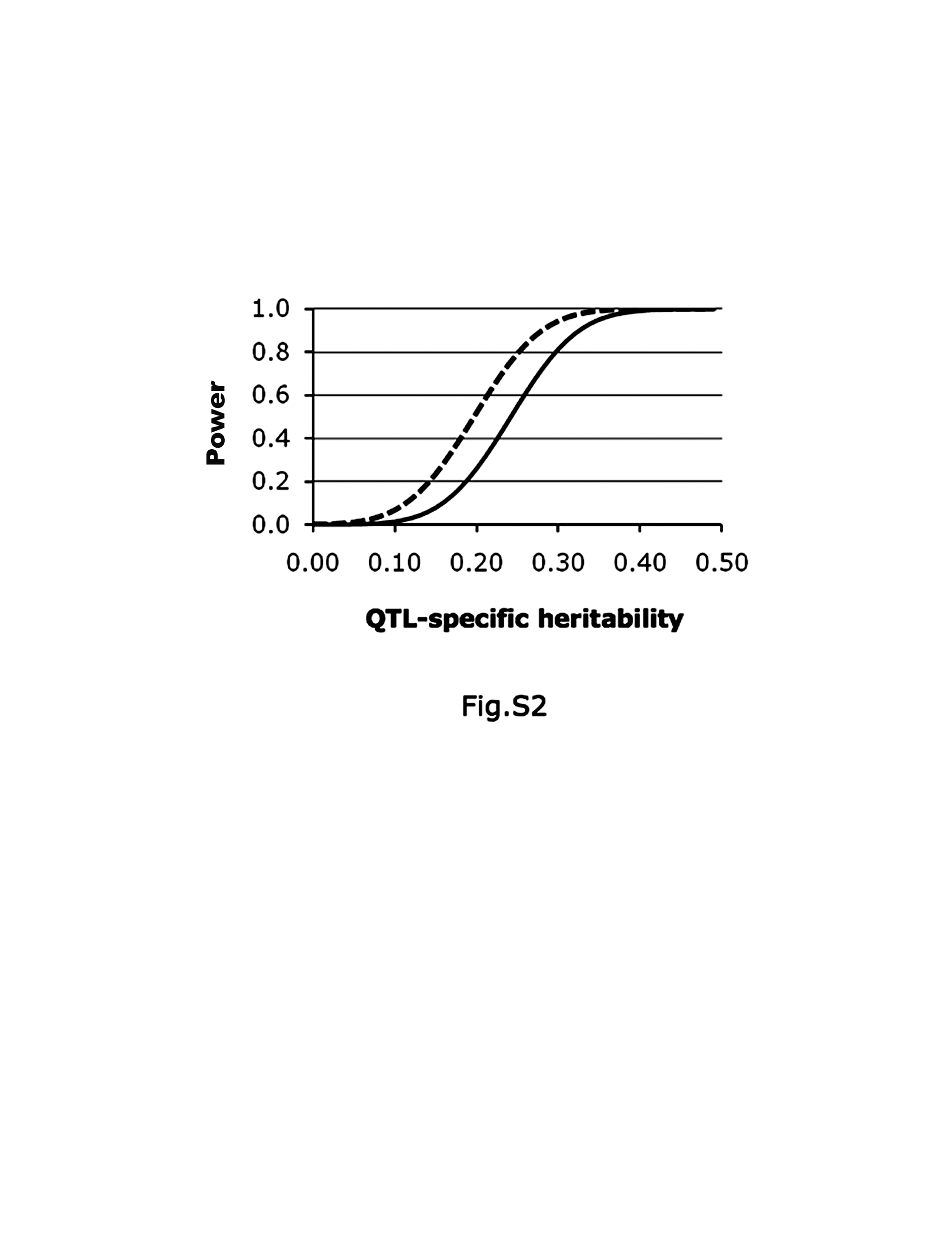

Supplement: Figure S2 — Power calculation of significant lod scores that could be obtained with the whole pedigree for traits of different heritability. Continuous line: lod score> = 3, broken line: lod score > = 2. The analysis was done with Solar (http://solar.sfbrgenetics.org/). (0.11 MB TIF) [file pone.0007554.s002.tif]
